# Hierarchy of Bioapatites

**DOI:** 10.3390/ijms23179537

**Published:** 2022-08-23

**Authors:** Andrzej Kuczumow, Mieczysław Gorzelak, Jakub Kosiński, Agnieszka Lasota, Tomasz Blicharski, Jacek Gągała, Jakub Nowak, Maciej Jarzębski, Mirosław Jabłoński

**Affiliations:** 1 ComerLab Dorota Nowak, Radawiec Duży 196, 21-030 Motycz, Poland; 2 Department of Orthopaedics and Rehabilitation, Medical University of Lublin, K. Jaczewskiego 8, 20-090 Lublin, Poland; 3 Chair and Department of Jaw Orthopedics, Medical University of Lublin, Chodźki 6, 20-093 Lublin, Poland; 4 Department of Orthopaedics and Traumatology, Medical University of Lublin, K. Jaczewskiego 8, 20-090 Lublin, Poland; 5 Department of Physics and Biophysics, Poznan University of Life Sciences, Wojska Polskiego 38/42, 60-637 Poznan, Poland

**Keywords:** bioapatites, precursors of bioapatites, hydroxyapatite, maturation of apatites, stoichiometry

## Abstract

Apatites are one of the most intensively studied materials for possible biomedical applications. New perspectives of possible application of apatites correspond with the development of nanomaterials and nanocompounds. Here, an effort to systematize different kinds of human bioapatites forming bones, dentin, and enamel was undertaken. The precursors of bioapatites and hydroxyapatite were also considered. The rigorous consideration of compositions and stoichiometry of bioapatites allowed us to establish an order in their mutual sequence. The chemical reactions describing potential transformations of biomaterials from octacalcium phosphate into hydroxyapatite via all intermediate stages were postulated. Regardless of whether the reactions occur in reality, all apatite biomaterials behave as if they participate in them. To conserve the charge, additional free charges were introduced, with an assumed meaning to be joined with the defects. The distribution of defects was coupled with the values of crystallographic parameters “*a*” and “*c*”. The energetic balances of bioapatite transformations were calculated. The apatite biomaterials are surprisingly regular structures with non-integer stoichiometric coefficients. The results presented here will be helpful for the further design and development of nanomaterials.

## 1. Introduction

The development of structures that range in size from 1 to 100 nm (nanoparticles, nanomaterials) or of submicron-sized structures brings new opportunities for biomedical applications [1]. One of the most important factors that are beneficial when using nanoparticles (NPs) is the possibility of designing and controlling the structural characteristic of the nanomaterial, which indicates further physicochemical properties [2]. Some of the most intensively studied nanomaterials for bone and dentin tissues are based on apatitic nanostructures [3]. For that reason, detailed information about apatite compositions and structures open new ways for new biomedical applications of apatite-based composites.

The substances, somewhat enigmatically, called biological apatites are essential structural materials of hard tissues in humans and generally vertebrate organisms. Bones, dentin, enamel, and dental cementum are all built of these kinds of apatite [4,5]. Bioapatites differ in a meaningful way from the mineral hydroxyapatite [6]. In comparison with geological hydroxyapatite, bioapatites are supplemented with Mg, Na and carbonates; they also involve acid phosphates and differ in mechanical features. The diffraction pattern is much more smeared [7], with some shifts of maxima of relevant lines. This indicates that bioapatites are poorly crystallized entities. Nevertheless, bioapatite material is not uniform in the organism. The substance from the enamel is substantially different from the ones included in bone and dentin. The latter two seem to be somewhat similar in chemical and structural composition, but not in a morphological sense. It was observed that bioapatites are devoid of hydroxyl groups, totally or in part [8,9]. The materials possess dynamical features, and the process of bones and teeth changes is called maturation, or sometimes aging [10]. Bone tissues are, in fact, very reactive, obeying permanent recrystallization by resorption–precipitation processes, e.g., the so-called remodeling is a kind of a game between the action of chemical analysis by osteoclasts followed by chemical synthesis made by osteoblasts [11]. It is totally different from the case of enamel. All hard tissues are heterogeneous structures, so the spatial location of the elements should be studied.

The precipitation process is arranged in a biochemical way. Nucleation inhibitors disappear, while the apatite ionic concentrations increase. In conditions of supersaturation, the sediment arrives either homogenously or on templates. Essentially, the process obeys all the rules of sedimentation, and inhibitors and accelerators play only a secondary role. There are many opinions about what is the first sedimented substance, from Posner et al.’s [12] assertion that it is amorphous calcium phosphate, to suggestions that it is very poorly crystallized apatite. So, in the biochemical environment, typical inorganic sedimentation of bioapatites occurs, either by extracellular processes in matrix vesicles or by an intracellular process in mitochondria [13,14]. It is why we can consider all the connections between apatites, bioapatites, and precursors in a uniform manner in our contribution. 

There are several analytical techniques that can be successfully applied to the apatite studies, such as spectrometric analysis, i.e., FTIR Raman or XRF (X-ray fluorescence) [15,16,17]. However, here, we focused on X-Ray diffraction and previously presented data by several researchers. One should estimate the possibilities of differentiation of results obtained for crystalline apatites [18] and amorphous entities [19]. To detect the crystallinity of samples quantitatively, FTIR analyses are frequently applied [20]. The purity of crystalline substances can be qualitatively controlled by comparing the similarity of the diffraction pattern of the sample with that of the standard [21]. Of course, apatites and bioapatites can be characterized further, e.g., by their crystallite sizes via scanning and transmission electron microscopies (SEM and TEM), small-angle electron scattering (SAXS) coupled with Porod analysis (however, this method is now mainly applied for tracing ion tracks resulting from irradiation) [22], and dynamic light scattering (DLS) [23]. This question is outside the main assumptions of our paper.

One of the essential questions while considering bioapatites is their stoichiometric formulae. Although there are many apparently rigorous chemical formulae trying to define bioapatite, e.g., this one by Legros [24,25]

Ca_10−(x−u)_(PO_4_)_(6−x)_ (HPO_4_^2−^ or CO_3_^2−^)_x_(OH^−^; F^−^, Cl^−^)_2−(x−2u)_ (1) with 0 ≤ x ≤ 2 and 0 ≤ 2u ≤ x,

in fact, in real measurements, the stoichiometric coefficients seem to be non-integer and nonstoichiometric. Driessens [26] suggested that it might result from the admixing of different crystallographic phases to the bioapatite treated as an agglomerate. This opinion is now rejected in the sense of looking for separate entities. Still, the deconvolution of FTIR spectra for nanocrystalline apatite in the ν4 PO_4_ range allowed for the discovery of several phosphate bands that did not belong to an apatitic environment [27,28].

Both mineral apatites and bioapatites adopt many inorganic additions. Mg [29], Na, and carbonates are among the most important minor components. They change both crystallinity and mechanical features of bioapatites. Their role in the transitions between particular biominerals will be shown in further detail in this text. Sr is another element that is important for the compactness of the bone and its remineralization [30]. Of course, such elements as Zn and Fe are also present in trace amounts, but they have no influence on what is considered in this study.

The origin of apatite materials in organisms is in the middle of active discussion. In general, four substances are considered to be precursors [31] of hard tissues: amorphous calcium phosphate (ACP) [17,32,33,34], β-tricalcium phosphate [35], octacalcium phosphate (OCP) [36,37,38,39,40], and magnesium whitlockite [41,42]. Among them, β-tricalcium phosphate with the addition of Mg^2+^ and whitlockite are hardly distinguishable by X-ray diffraction (XRD) patterns [35]. The questions of whether they are involved in the reaction of forming biomaterials, which ones are participating in this process, and what the route of such a reaction is, are all open.

The main aim of this contribution is to answer the following questions: whether all apatite biomaterials are coupled to each other; if so, what is the sequence of these biomaterials; what kind of links join the biomaterials with their precursors; whether there exist reactions linking particular bioapatites; and how much energy is necessary to perform such transformations.

## 2. Results

### 2.1. Boundary Compounds

In line with the aim of this study, we want to present the possible sequence of inorganic chemical entities that participate in the formation of hard tissues. At first, one must establish the boundary substances in this series. From the initial side, the precursor substances of bioapatite [43] should be considered, i.e., amorphous calcium phosphate (ACP), β-tricalcium phosphate, octacalcium phosphate (OCP), and magnesium whitlockite. Most probably, the synthesis of bioapatite starts with those compounds. On the other side, hydroxyapatite is a final point of reference in the sequence of the relevant apatites. In between, biomaterials such as dentin, bone, and enamel should be located.

Starting with the precursor substances, one should consider what they look like and whether they can be transformed into hydroxyapatite in one step. This is illustrated in the following chemical equations:

Ca_8_(HPO_4_)_2_(PO_4_)_4_*5H_2_O + 2Ca(OH)_2_ → Ca_10_(PO_4_)_6_(OH)_2_ +7H_2_O (2)
(octacalcium phosphate → hydroxyapatite)

Ca_9_Mg(PO_4_)_6_(HPO_4_) + Ca(OH)_2_ → Ca_10_(PO_4_)_6_(OH)_2_ + MgHPO_4_ (3) (magnesium whitlockite → hydroxyapatite)

Ca_9_(PO_4_)_6_ + Ca(OH)_2_ → Ca_10_(PO_4_)_6_(OH)_2_ (4) (amorphous apatite → hydroxyapatite)

Transformation described by Equation (4) based on [44]. One can see that each time the reaction with Ca(OH)_2_ occurs, it suggests an alkaline environment. Here, we introduced Ca(OH)_2_ as the entity, not suggesting that this substance works as such. However, the total balance of ions indicates that their participation is necessarily accompanied by these conditions. The molecular notation for Ca(OH)_2_ is given later in this article. In addition, the delivery of Ca^2+^ and hydroxyl ions is necessary to obtain the correct composition of hydroxyapatite. If there exist any intermediate phases between the precursors and hydroxyapatite, they have to be supplemented with calcium and hydroxyl ions. By assumption, the more deficient the biomaterials in both kinds of ions, the less mature their phases.

How can we prioritize the above-mentioned precursors? It is easy to note that the preceding step before the action presented in Equation (4) can be shown as:

Ca_8_(HPO_4_)_2_(PO_4_)_4_*5H_2_O + Ca(OH)_2_ → Ca_9_(PO_4_)_6_ + 7H_2_O (5)

Next, we can continue with Equation (4) to obtain hydroxyapatite. Thus, octacalcium phosphate is a precursor substance for amorphous apatite. Similarly:

Ca_8_(HPO_4_)_2_(PO_4_)_4_*5H_2_O + Ca(OH)_2_ + Mg(HPO_4_) → Ca_9_Mg(PO_4_)_6_(HPO_4_) + 7H_2_O (6)

Here, one must add Equation (2). Octacalcium phosphate is also the precursor for magnesium whitlockite, and in general, it is the basic precursor for hydroxyapatite.

Since there are suggestions [45,46,47] that brushite Ca(HPO_4_)*2H_2_O also belongs to the category of precursors of bioapatites, we can give an idea of potential reaction leading to form octacalcium phosphate from this substance:

8Ca(HPO_4_)*2H_2_O + 3H_2_O → Ca_8_(HPO_4_)_2_(PO_4_)_4_*5H_2_O + 2H_3_PO_4_ (7)

Indeed, brushite is observed both in the processes of extracellular formation of bioapatites and in the processes of intracellular fabrication [48].

The above reaction can be considered hydrolysis in an alkaline environment joined with dephosphorylation. In such an approach, brushite would be the most primary precursor, but it is outside the scope of our paper.

### 2.2. Basic Phosphate Biomaterials

It is well-known that the composition of natural bioapatite in bone, dentin, and enamel can be only presented with the use of experimental stoichiometric coefficients, far away from integer numbers. However, efforts have been made to rationalize the chemical formulae of bioapatites, as those from the paper by Combes et al. [49]:

Human bone apatite: Ca_8.1_Mg_0.2_(PO_4_)_4.3_(HPO_4_)_0.5_(CO_3_)_1.2_(OH)_0.3_ (8)

Human dentine apatite: Ca_8.0_Mg_0.4_(PO_4_)_4.4_(HPO_4_)_0.7_(CO_3_)_0.9_(OH)_0.4_ (9)

Human enamel apatite: Ca_8.8_Mg_0.1_(PO_4_)_4.9_(HPO_4_)_0.6_(CO_3_)_0.5_(OH)_0.9_ (10)

**Figure 1:**
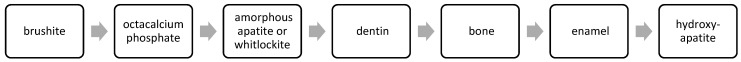
Postulated evolution of substances changes.

However, at first, they were not very rigorous and did not involve sodium, which also had to be taken into consideration. Nevertheless, such formulae allow us to draw some conclusions: we have three main hard-tissue-forming environments in the body—the first one leading to the formation of dentin, the next one to bone, and the third one leading to enamel. Thus, it suggests the following sequence of substances presented in Figure 1.

The last conclusions from the above formulae are those concerning the hydroxyl group. Since there is a great discrepancy in the contents of this group in the bioapatite molecules, we used the amounts from Equations (8)–(10) to supplement the formulae for bone, dentin, and enamel in our further calculations. The coefficients by Combes are in reasonable accordance with the data by Kolmas [50] derived from measurements (0.72 for the enamel, and 0.18 for the dentin). Taking all this together, we prefer the values 0.3 for dentin, 0.4 for bone, and approximately 0.7 for enamel.

The important aim is to make a rigorous hierarchy of materials in the sense of maturation. From what has been said so far, it can be concluded that more mature compounds should involve greater amounts of Ca and hydroxyl group (see also formulae presented in Equations (8)–(10)). The greater crystallite sizes also indicate a greater maturity of crystals [51]. From the data of Dorozhkin [4], it can be concluded that bone apatite is a little more matured than dentin apatite.

### 2.3. Formulae

We found some data available in the literature about the composition of apatite in bone, dentin, and enamel. As a rule, the data that concerned the measurements collected in one measurement system were preferred, but some individual pieces of data of significant credibility were also taken into account. The data were uniformized by summarizing the molar amounts of cations (∑(Ca + Mg + Na + K)) and normalizing them to the value of 10, which is the stoichiometric coefficient concerning the presence of Ca in pure hydroxyapatite. The information concerning carbonates demands taking different locations of the ion instead of phosphate (substitution B, denoted as BCO_3_) and inside the tetrad channel (substitution A, ACO_3_). In such an approach, the results are as follows. Equation (11) was made according to Dorozhkin [4], Le Geros [52], Daculsi et al. [53]. Equation (12) based on the Skinner work [54], and Equation (13) was done according to Le Geros [55].

For bone:

Ca_9.176_Mg_0.539_Na_0.275_K_0.011_(HPO_4_)_0.009_(PO_4_)_5.772_(BCO_3_)_0.286_(OH)_0.011_Cl_0.039_F_0.032_(ACO_3_)_0.697_ (11)

Ca_9.331_Mg_0.217_Na_0.456_(HPO_4_)_0.01_(PO_4_)_5.436_(BCO_3_)_0.456_(OH)_1.096_(ACO_3_)_0.607_*1.628H_2_0 (12)

Ca_9.34_Mg_0.197_Na_0.44_K_0.01_(PO_4_)_5.39_(BCO_3_)_0.45_Cl_0.037_(ACO_3_)_0.65_ (13)

We use the version of Skinner, corrected for the amount of OH (coefficient 0.4), enriched in Cl and F, neglecting the water.

For dentin: 

Furthermore, for dentin Equation (14) was proposed according to Dorozhkin [4], and Equation (15) based on the study presented by Le Geros [55].

Ca_9.182_Mg_0.535_Na_0.273_K_0.01_(HPO_4_)_0.01_(PO_4_)_5.723_(BCO_3_)_0.283_(OH)_0.01_F_0.031_(ACO_3_)_0.701_ (14)

Ca_9.18_Mg_0.46_Na_0.38_K_0.1_(PO_4_)_5.69_(BCO_3_)_0.48_(ACO_3_)_0.487_ (15)

We use the version of Dorozhkin, corrected for the amount of OH (0.3)

For enamel:

The Equations (16)–(21) were proposed according to Dorozhkin work [4], Hendricks and Hill [56], Moreno and Aoba [57]), Elliott [36], and Patel and Brown [58] respectively.

Ca_9.562_Mg_0.188_ Na_0.23_K_0.021_(HPO_4_)_0.001_(BCO_3_)_0.251_(PO_4_)_5.965_(OH)_0.0026_ Cl_0.084_F_0.01_(ACO_3_)_0.354_ (16)

Ca_9.703_Mg_0.184_Na_0.113_(PO_4_)_5.806_(BC0_3_)_0.113_(OH)_1.577_(ACO_3_)_0.348_(H_2_0)_0.471_ (17)

Ca_10_(HP0_4_)_0.184_(PO_4_)_6.06_(C0_3_)_0.622_(0H)_0.115_ (18)

Ca_9.578_Mg_0.097_Na_0.313_K_0.011_(HPO_4_)_0.303_(BCO_3_)_0.443_(PO_4_)_5.74_(OH)_0.757_Cl_0.086_(ACO_3_)_0.54_ (19)

Ca_9.519_Mg_0.167_Na_0.303_K_0.01_(PO_4_)_5.858_(BCO_3_)_0.313_(OH)_1.328_Cl_0.073_F_0.01_(ACO_3_)_0.325_ (20)

We use the version of Elliott, strongly confirmed in our previous studies presented in [59].

### 2.4. Potential Transformations from Octacalcium Phosphate to Hydroxyapatite

For the conservation of charge, we introduced additional charges to balance both sides of the equations, and we denoted them with squares and the sign of charge. The simplest explanation for them is that the additional charges result from crystallographic defects in biomaterials. The calculation is made from the end, i.e., to fix 0 value for the additional charges on mineral hydroxyapatite, which has no hydrated layer with a non-apatitic environment [5,60]. It seems to be more reasonable than to fix this value to precursor substances, which are chemically active (see Table 1).

As it was introduced earlier, the designation BCO_3_ means traditional carbonates which participated in the so-called substitution B for phosphates, while ACO_3_ means not only the substitution A in the tetrad channel of apatite, but also all other carbonates joined to the apatite, except those participating in substitution B. We recognize that the number of B-substituted carbonates is equal to the total number of Na and K ions (except in the enamel case, where we invoke Elliott’s data). In general, carbonates are delivered with body fluids, are included in a hydrated layer 1–2 nm thick on the surface of bioapatites, and their level can be controlled with carbonic-anhydrase enzymes.

The balance of Ca^2+^ in all equations is 2.000 (we invoke here Equation (2) as a limiting standard), the sum of hydroxyl groups is 2.001, and Mg is 1.000. Magnesium whitlockite and bone presented an excess of positively charged vacancies, while enamel and dentin presented an excess of negatively charged ones. One can claim that the problem of unbalanced charges was shifted by us to the whitlockite and bioapatite. However, we can reply that it is a well-known fact that the core crystals of bioapatites are surrounded by a structured surface hydrated layer supplying crystals with necessary ions, and that they can balance the charges to 0 [5,61,62,63,64,65].

Please note that numerous ions of Mg and phosphates are liberated in the reaction presented in Equation (23). In connection with NH_4_^+^ ions, it can lead to the secretion of struvite Mg(NH_4_)PO_4_, one of the main components of urinary stones. It is a side and marginal effect.

One might ask how to stop the crystallization of bioapatite at any stage, without the transformation in fully crystallized hydroxyapatite. Here, the natural presence of inhibiting citrate ions in some bioapatites seems to be the answer [66,67,68]. Even if hydroxyapatite crystals grow, their size is then decreased and controlled [69], but this aspect is outside the scope of our paper.

## 3. Discussion

The effort to extract the hierarchy of bioapatite and their precursors from the existing data gave interesting but only partially expected results. At first, it was possible to present chemical equations describing the passing from the so-called precursors to the final stage, i.e., hydroxyapatite. Among three precursor substances, the octacalcium phosphate was a real precursor, from which one could go to the secondary precursors—amorphous calcium phosphate and whitlockite. However, even the octacalcium phosphate could be originated from the brushite (Equation (7)). The reactions were very simple ones, essentially with Ca(OH)_2_. Next, it was proved that both secondary precursors can be transformed into hydroxyapatite via reaction with Ca(OH)_2_ as well. Perhaps it occurs indirectly, but the chemical equations fulfil all conditions to become so. This reaction with Ca(OH)_2_ was very important, and it occurred in each stage of transformations. Please pay attention to the fact that we do not suggest that the tissues built of bioapatite form themselves one from another by described reactions, according to the location in the sequence of transformations. We suggest only that the tissues possible to obtain are all lying in the postulated series of sequences.

In our approach, the differentiation between dentine and bone material is very clear, with the visible portion of Ca(OH)_2_ that must be used to transform the first material into the other one. Dentin is a less advanced material than bone. Even the kinds of charges attributed to the defects are different in both tissues. 

**Figure 2:**
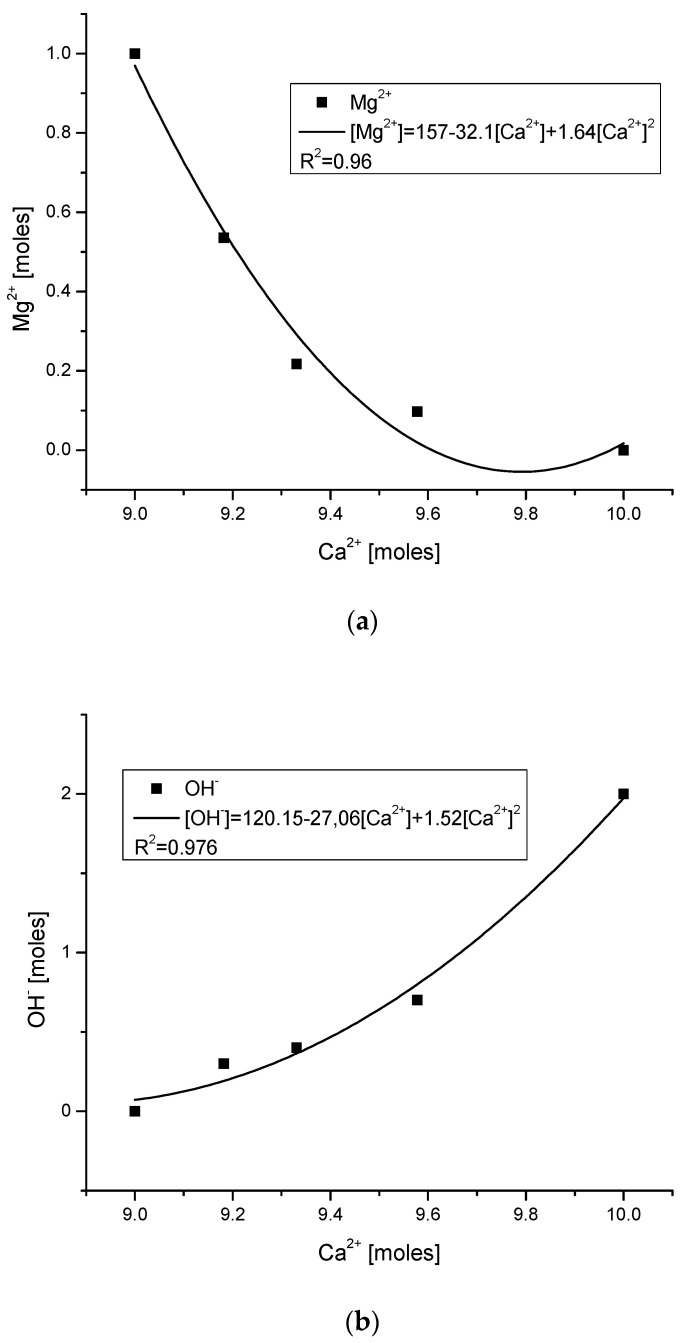

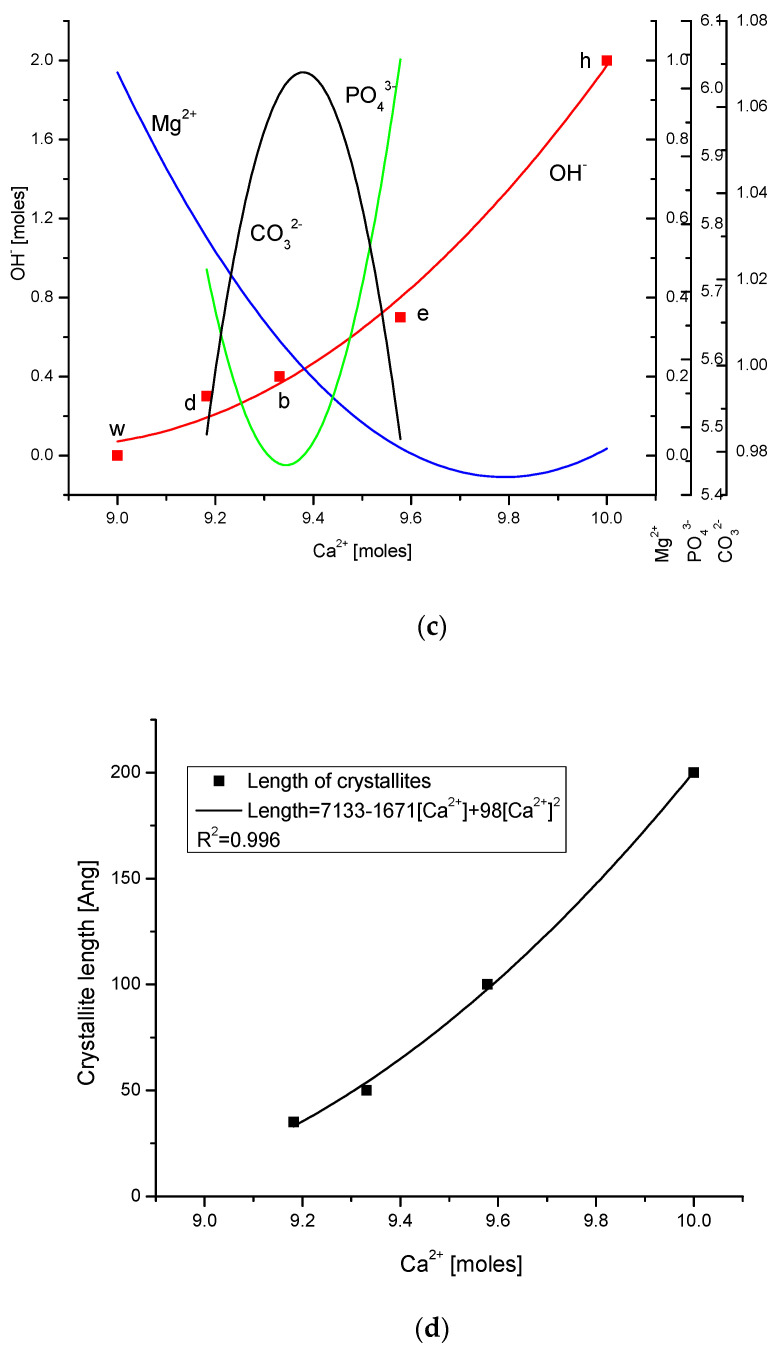

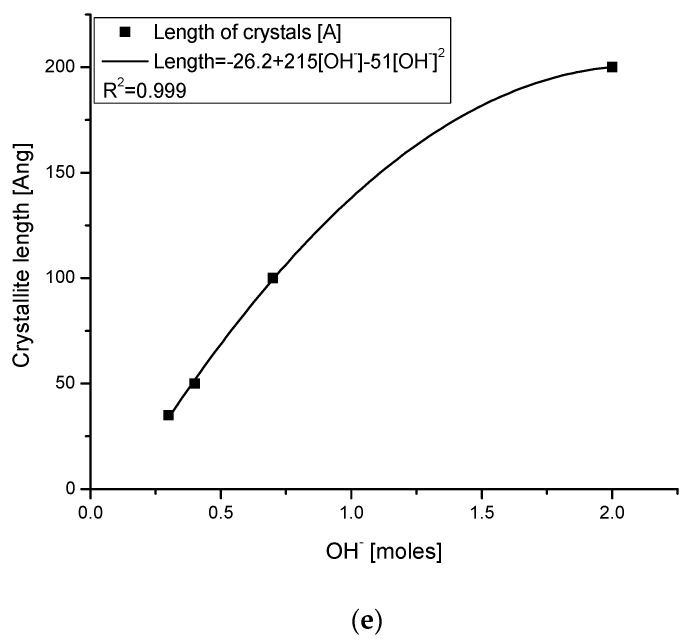
(**a**,**b**) Relationships between Ca^2+^ and Mg^2+^ or OH^−^ ions in bioapatites; (**c**) tendency of changes inside bioapatites during transformations; relationship between the length of plate-like crystallites and (**d**) Ca^2+^ ion molar contents; (**e**) OH^−^ ion contents. (Letters w, d, b, e, and h mean whitlockite, dentin, bone, enamel, and hydroxyapatite, respectively.) Please notice that the localization of points on the Ca axis corresponds to particular phases of bioapatites.

The hierarchical sequence of bioapatites makes it clear that less advanced materials have nearly no hydroxyl groups. The precursory substances include no hydroxyl group, while the hydroxyapatite involves two groups. On the way from octacalcium phosphate to hydroxyapatite, the hydroxyl groups arrived gradually. A wonderful regularity of ionic variability during a hypothetical passage from magnesium whitlockite to hydroxyapatite is shown in Figure 2c. Please note the decline in Mg contents, the growth in hydroxyl amount, and the inverse behavior of phosphate and carbonate ions.

One of the interesting consequences of our approach is the dependence of crystallite lengths (as cited by Dorozhkin [4]) on the molar contents of Ca and hydroxyl ions. The dependencies are paraboloidal (see Figure 2d,e). However, we must remember that the crystallites here are those observed and measured by SEM or TEM, which cannot be confused with the coherence volumes calculated according to the Scherrer equation.

**Figure 3:**
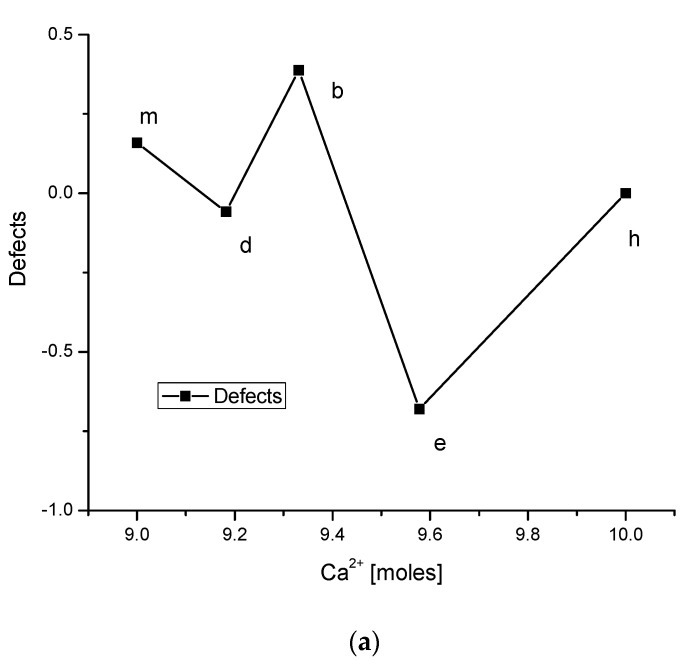

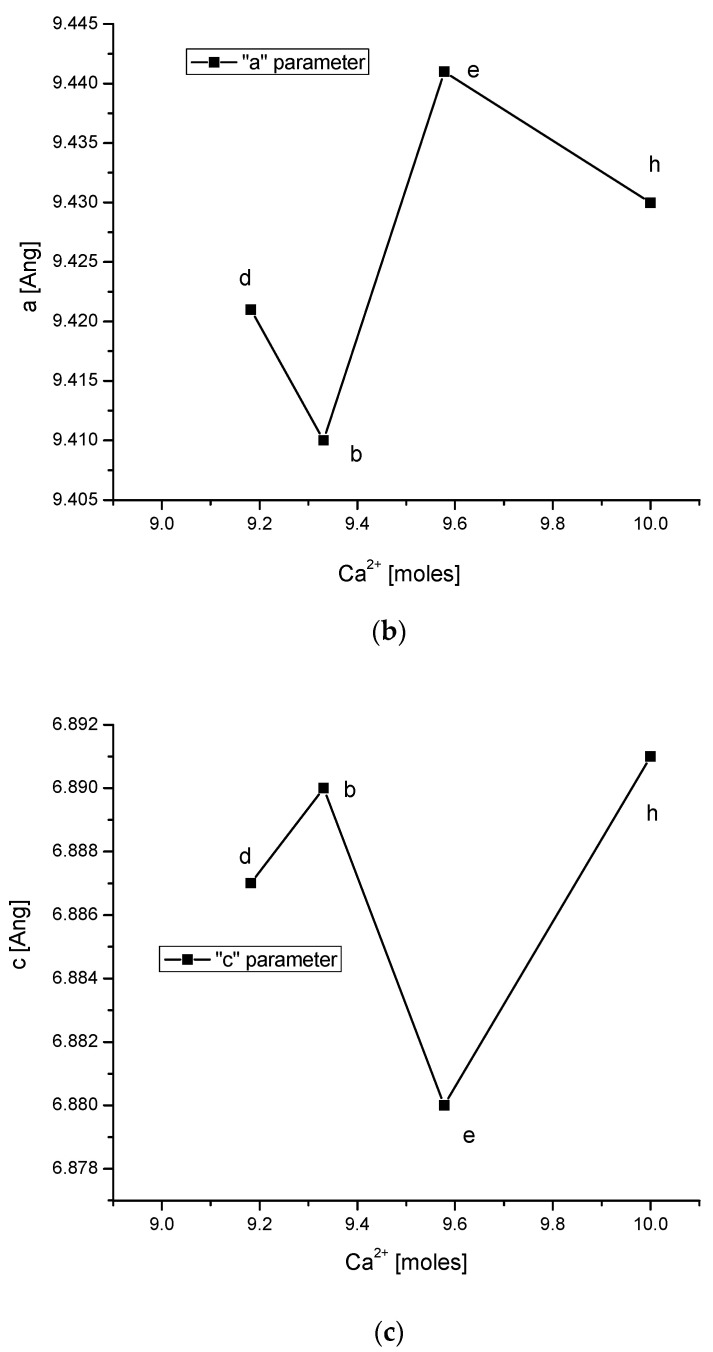

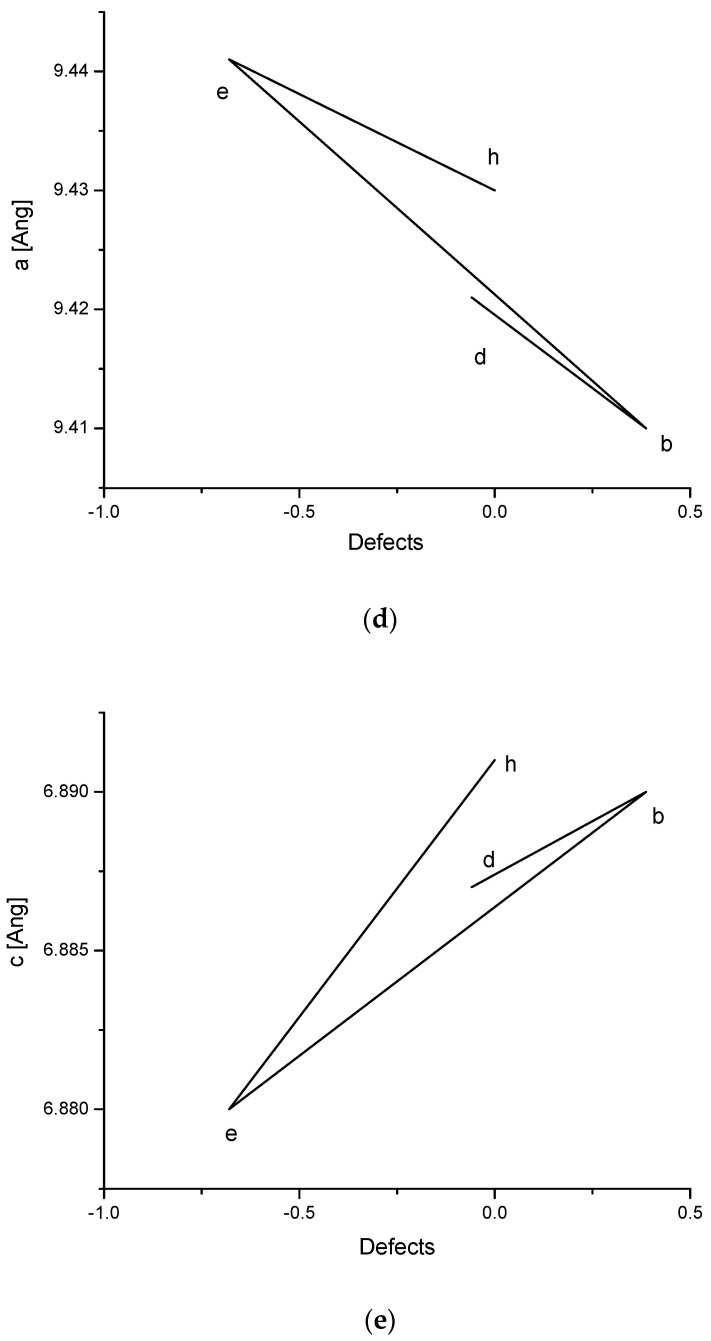
(**a**) Distribution of charges (defects) in different phases of bioapatites; (**b**) distribution of values of crystallographic parameter “*a*”; (**c**) the same for parameter “*c*”; (**d**,**e**) relationships of parameter “*a*”, “*c*” on charge of defects. Letters denote, respectively: b—bone; d—dentin; e—enamel; h—hydroxyapatite. (The solid lines linking points in the figures are eye guides rather than real relationships).

Very interesting relationships arose between the distributions of ionic defects and “*a*” and “*c*” crystallographic parameters (see Figure 3a,b). The variability in charge distribution is proportional to the distribution of the “*c*” parameter and indirectly proportional to the distribution of the “*a*” parameter. If one presents the relationships between particular crystallographic parameters and the value of charge, the results are surprising. The sets of crystallographic parameters (*a*, *c*) are relatively close for dentin and hydroxyapatite. In between, the sets of parameters (*a*, *c*) deviate, with smaller *c* and greater *a* for bone, and the opposite trend for enamel.

The important question is how much energy is necessary to transform the particular phases of bioapatite one into another. The problem can be solved easily. We use a specific form of Braggs law:

n*12.4/E = 2d*sin Θ (27)

where the value of “*d*” results from

1/*d*^2^ = 4/3(h^2^ + hk + k^2^)/*a*^2^ + l^2^/***c***^2^ (28)

and describes the simplest crystallographic cell when we introduce (1,1,1) indices.

If one differentiates Equation (27), assuming that *n* = 1, angle Θ is a constant measured value for a given case, and that energy E is then dependent on the value of dimension “*d*”, we have (but keeping the notations expressed in differences Δ, not in differentials δ):

ΔE = −6.2*Δ*d*/(sin Θ*d^2^) (29)

Then, using the crystallographic data from Dorozhkin [4], one can easily calculate that the transformation of dentin material into bone material (Equation (23)) releases 5.3 eV per crystallographic unit cell, bone into enamel (Equation (24)) demands 14.3 eV, and enamel into hydroxyapatite (Equation (25)) releases 1.9 eV. The results emphasize the fact that there is a relatively great difference between bone and enamel and a relatively small difference between enamel and hydroxyapatite. The transformations between dentin and bone and enamel into hydroxyapatite should occur spontaneously. If it does not occur, that is probably due to the fact that the reactions are occurring in different locations of the body. Particles of pure hydroxyapatite are devoid of an active surface layer [70], and the substance becomes passive since a potential reaction depends on the slow diffusion rate in a solid state.

Summarizing the discussion, we must emphasize once more that we do not suggest that the reactions mentioned in general in Equation (2) are actually going on. However, all the numerical and illustrative (Figure 1 and Figure 2) proofs show that the bioapatites behave as though the reactions really do occur, in the most rigorous manner. Then, our suggestions will probably have practical aspects. Attention was paid to the role of Ca(OH)_2_ in passing from one step in our sequence to another one. Perhaps this harsh reaction should be taken into account in artificial syntheses of bioapatites. However, we treat the formulation with Ca(OH)_2_ as an illustration of stoichiometry conservation only. Similarly, the energetic balance of the transformations (given by Equation (29)) is of importance in syntheses. In addition, we suppose that Figure 2c should be very informative when estimating any imitations of natural biopatites.

## 4. Materials and Methods

This study was inspired by the previous research described in detail in [71]. Here, a detailed discussion of human enamel was presented, where the spatial distributions of elements and crystallographic parameters “*a*” and “*c*” were shown. In these studies, electron probe microanalysis (EPMA) and X-ray diffraction (XRD) were applied. The variability of the enamel on its way from the contact of enamel with air, up to the boundary of enamel with dentin, was surprisingly regular, obeying the exponential relationship along the distance [71]. This suggests that bioapatites are much more ordered and stoichiometric than has been suggested in many papers. We decided to widen the search on other bioapatites and the substances called precursors for bioapatites.

We used the available scientific literature data concerning bone, dentin, and enamel and the data on hydroxyapatite that presented this substance in permissible hexagonal structure [72,73]. Additionally, data on so-called precursors of bioapatites were found in the literature. We used only information considered very reliable and obtained from recognized laboratories. Rather unexpectedly, in a positive sense, these mentioned data were well-synchronized to each other, and we could select the most appropriate ones for our aims. Nevertheless, some corrections were necessary, concerning, e.g., the number of hydroxyl groups and the partition of carbonate ions between those existing as B- and A-substituted. The data on the HPO_4_^2−^ group were generally not very reliable [31].

## 5. Conclusions

We managed to make an order in a sequence of bioapatites in a series: brushite → octacalcium phosphate → amorphous phosphate, magnesium whitlockite → dentin → bone → enamel. The postulated relevant chemical reactions were given for each stage of the above transformations. The most important was a formal reaction of substances with Ca(OH)_2_. The essential role of the Ca, Mg, and OH group in determining such a sequence of biomaterials was clearly proven. Moreover, the variability of the long axis of microscopically measured apatite crystallites was very similar to the variability of Ca and OH ions. To overcome the inconsistency in the conservation of charges, additional free charges were introduced. We assigned to them the meaning charges resulting from defects. The distribution of defects was generally consistent with the variability of crystallographic parameters “*a*” and “*c*”. Finally, we calculated the energetic effects of the transformations of bioapatites. Summing up, the bioapatites turned out to be surprisingly logical and ordered structures, with all processes leading to the formation of any form of bioapatite in the human organism strictly coupled, perhaps as a result of enzymatic biomineralization [74].

## Figures and Tables

**Table 1:** Postulated passages from octacalcium phosphate to hydroxyapatite via bioapatites.

Transformation Formula	Octacalcium Phosphate, Amorphous Apatite → Whitlockite	Equation No.
	Ca_8_(HPO_4_)_2_(PO_4_)_4_*5H_2_O + Ca(OH)_2_ +Mg(HPO_4_) → Ca_9_Mg(PO_4_)_6_(HPO_4_) + 7H_2_O or Ca_9_(PO_4_)_6_*nH_2_O + Mg(HPO_4_) → Ca_9_Mg(PO_4_)_6_(HPO_4_) + nH_2_O	(21) (22)
	**Whitlockite → dentin**	
	Ca_9_Mg(PO_4_)_6_(HPO_4_)□^+^_0.158_ +0.15Ca(OH)_2_ + 0.032Ca^2+^ + 0.273Na^+^ + 0.01K^+^ + 0.984CO_3_^2−^ + 0.031F^−^ → Ca_9.182_Mg_0.535_Na_0.273_K_0.01_(HPO_4_)_0.01_(PO_4_)_5.723_(BCO_3_)_0.283_(OH)_0.3_F_0.031_(ACO_3_)_0.701_□^−^_0.059_ + 0.465Mg(HPO_4_) + 0.273(PO_4_^3−^) + 0.525(HPO_4_^2−^)	(23)
	**Dentin → bone**	
	Ca_9.182_Mg_0.535_Na_0.273_K_0.01_(HPO_4_)_0.01_(PO_4_)_5.723_(BCO_3_)_0.283_(OH)_0.3_F_0.031_(ACO_3_)_0.701_□^−^_0.059_ + 0.05Ca(OH)_2_ + 0.099Ca^2+^ + 0.183Na^+^ + 0.080CO_3_^2−^ → Ca_9.331_Mg_0.217_Na_0.456_K_0.01_(HPO_4_)_0.01_(PO_4_)_5.436_(BCO_3_)_0.456_(OH)_0.4_ F_0.031_(ACO_3_)_0.607_□^+^_0.387_ + 0.318Mg^2+^ + 0.287PO_4_^3−^	(24)
	**Bone → enamel**	
	Ca_9.331_Mg_0.217_Na_0.456_K_0.01_(HPO_4_)_0.01_(PO_4_)_5.436_(BCO_3_)_0.456_(OH)_0.4_F_0.031_(ACO_3_)_0.607_□^+^_0.387_ + 0.179Ca(OH)_2_ + 0.068Ca^2+^ + 0.001K^+^ + 0.293(HPO_4_)^2−^ + 0.304(PO_4_)^3−^ → Ca_9.578_Mg_0.097_Na_0.313_K_0.011_(HPO_4_)_0.303_(BCO_3_)_0.443_(PO_4_)_5.74_(OH)_0.757_Cl_0.086_F_0.01_(ACO_3_)_0.54_□^−^_0.68_ + 0.12Mg^2+^ + 0.143Na^+^ + 0.008CO_3_^2−^ + 0.021F^−^	(25)
	**Enamel → hydroxyapatite**	
	Ca_9.578_Mg_0.097_Na_0.313_K_0.011_(HPO_4_)_0.303_(BCO_3_)_0.443_(PO_4_)_5.74_(OH)_0.757_ Cl_0.086_F_0.01_(ACO_3_)_0.54_□^−^_0.68_ + 0.422Ca(OH)_2_ + 0.399OH^−^ → Ca_10_(PO_4_)_6_(OH)_2_ + 0.054Mg^2+^ + 0.313Na^+^ +0.011K^+^ + 0.983CO_3_^2−^ + 0.086Cl^−^ + 0.01 F^−^ + 0.043MgHPO_4_	(26)

□ means defect.

## Data Availability

Not applicable.
